# Endoscopic Sciatic Neurolysis for Deep Gluteal Syndrome: A Systematic Review

**DOI:** 10.7759/cureus.23153

**Published:** 2022-03-14

**Authors:** Sreenivasulu Metikala, Vivek Sharma

**Affiliations:** 1 Orthopaedic Surgery, Virginia Commonwealth University School of Medicine, Richmond, USA

**Keywords:** arthroscopic sciatic nerve decompression, endoscopic sciatic nerve release, endoscopic approach, piriformis muscle syndrome, endoscopic sciatic neurolysis, endoscopic sciatic nerve decompression, sciatic nerve entrapment, piriformis syndrome, deep gluteal syndrome, deep gluteal pain syndrome

## Abstract

Deep gluteal syndrome (DGS) is an underdiagnosed condition caused by an extra-spinal entrapment of the sciatic nerve in the deep gluteal space. Symptomatic patients who fail conservative treatment require surgical decompression of the nerve either by an open or endoscopic approach. In recent times, there has been an increasing trend towards minimally invasive surgery performed with endoscopic techniques. This systematic review aimed to assess the effectiveness of endoscopic sciatic nerve decompression in the management of DGS. A comprehensive search of the PubMed, Web of Science, Cumulated Index to Nursing and Allied Health Literature (CINAHL), and SPORTDiscus databases were performed on January 3, 2022. All English-language clinical studies on DGS treated with endoscopic surgical decompression were included. The initial search criteria identified 145 articles, of which four studies were available for the final review. There was one level III evidence, while the remaining three were level IV, comprising 144 patients with a mean age of 46 years. The Coleman methodology score (CMS) was utilized to assess the quality of the studies and the mean score was 62 (range, 52 to 71). The presence of fibrovascular bands and bursal tissue was the most common cause of DGS, followed by musculotendinous structures. The average follow-up of the included studies was 26.3 months (range, 12 to 32 months). Less favorable outcomes were seen in patients with major traumatic sciatic neuropathies after fractures or open reconstructive hip surgeries. Conversion to formal open surgery was recorded in one case of DGS caused by sciatic nerve schwannoma due to poor endoscopic access. One patient developed postoperative recurrent sciatic nerve entrapment due to a foreign body reaction requiring an open decompression. Overall, the available studies reported a high degree of clinical success with a low rate of complications, albeit no high-quality studies could be identified.

## Introduction and background

**Figure 1:**
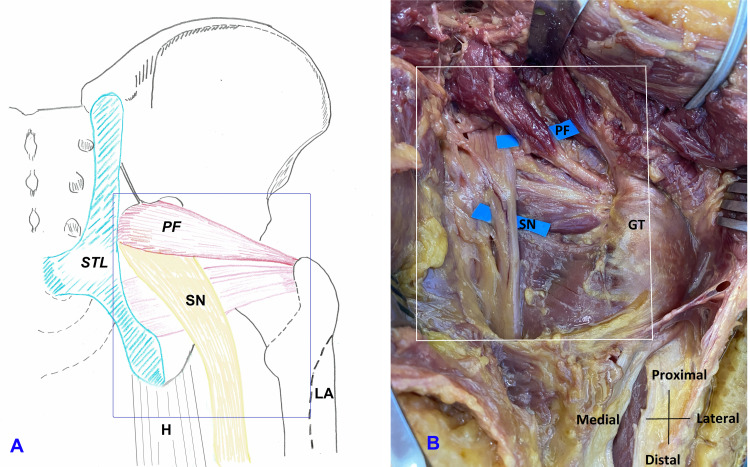
Boundaries of the right deep gluteal space. A: Illustration. B: Anatomic dissection STL: Sacrotuberous ligament, PF: Piriformis, SN: Sciatic nerve, H: Hamstring tendon, LA: Linea aspera, GT: Greater trochanter

Deep gluteal syndrome (DGS) describes non-discogenic and extra-pelvic entrapment of sciatic nerve in the deep gluteal space, also called subgluteal space between the middle and deep gluteal aponeurotic layers [1,2]. The anatomic boundaries of deep gluteal space include gluteus maximus posteriorly, femoral neck with greater and lesser trochanters anteriorly, sacrotuberous ligament medially, and linea aspera laterally (Figure 1).

The space is limited proximally by the inferior margin of the greater sciatic notch, while the origin of hamstrings at ischial tuberosity marks the distal extent. The sciatic nerve, the main content of deep gluteal space, leaves the pelvis via the greater sciatic notch and enters this area at the inferior margin of the piriformis muscle. The nerve then courses distally posterior to the short external rotator muscle complex and exits towards the posterior thigh through the ischiofemoral tunnel between the greater trochanter and ischial tuberosity. While in the deep gluteal space, the nerve enjoys substantial mobility up to 28 mm to accommodate pelvic motion and hip joint movements in all planes [3]. Therefore, any structure or abnormality in the deep gluteal space impeding this normal nerve excursion may result in DGS. The common pathologies include fibrous and fibrovascular bands, piriformis syndrome, short external rotator complex (triceps coxae), hamstring muscles, gluteal muscles or tendons, vascular abnormalities, and space-occupying tumors [2,4-7]. Though piriformis syndrome was considered synonymous with DGS in the past, it has now been recognized that piriformis syndrome is only a subgroup of DGS. Other potential causes include overuse-related conditions, high-energy trauma such as acetabular fractures or posterior hip dislocations and postoperative scarring, hematoma, protruding hardware, or heterotopic ossification [8-10].

Clinically, DGS presents with a variable constellation of symptoms in the adult population similar to radicular pain of spine etiology, thus posing a diagnostic challenge. Unilateral buttock pain radiating distally to the posterior thigh or groin with an inability to sit for more than 30 minutes is the most common presentation. A typical antalgic sitting position, bearing weight on the unaffected ischium, is frequently seen in the affected individual [4]. The physical examination includes sciatic notch tenderness with positive flexion-adduction-internal rotation (FADIR) test, active piriformis, and seated piriformis stretch tests [6,11,12]. Plain radiographs, ultrasound (US), and magnetic resonance imaging (MRI) are the standard investigations in the diagnostic work-up of DGS. Electromyography (EMG) and nerve conduction study (NCS) are of doubtful value, which may demonstrate conduction abnormalities and denervation potentials. Magnetic resonance arthrogram (MRA) is indicated when a concomitant labral tear is suspected. The MRI scan of the lumbosacral spine is often necessary to exclude a spine disease. Ultimately, MRI scan has been established as the imaging modality of choice in DGS evaluation. The US-guided steroid plus local anesthetic injections are frequently performed for diagnostic decision-making and differentiate from other potential pelvic-related sources of pain [13-15].

**Figure 2:**
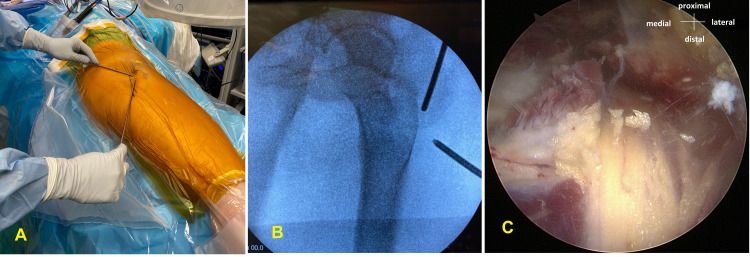
Endoscopic technique of right sciatic neurolysis. A: Lateral decubitus position with planned proximal and distal trochanteric portals, B: Fluoroscopic image for localization, C: Endoscopic image of the sciatic nerve

Nonsurgical treatment, including activity modifications, oral nonsteroidal anti-inflammatories, and physical therapy (PT) for at least six weeks, is an appropriate first-line approach in the management [1,2]. The PT regimen should include gentle stretching exercises of external rotators, sciatic nerve glides, and hip circumduction exercises. Ultrasound therapy and electrical stimulation techniques can be particularly beneficial to address acute symptoms. The other adjuncts consist of ultrasound-guided hydrodissection adhesiolysis [15,16] and steroid-local anesthesia injections. As a general rule, patients with symptoms recalcitrant to conservative measures are considered for surgical decompression of the sciatic nerve through an open or endoscopic technique. Historically, reasonable outcomes have been obtained after the open surgical approach [17-20]. The endoscopic approach was first employed by Dezawa et al. for piriformis muscle release under local anesthesia using a 4-mm arthroscope with a 30-degree viewing angle [21]. Later, Ilizaliturri et al. presented an endoscopic technique for iliotibial band release in 11 patients with external snapping hip syndrome [22]. The first description of endoscopic sciatic nerve decompression using 70-degree standard and long arthroscopes was published by Martin et al. in a series of 25 patients with DGS [12]. It is typically performed under fluoroscopic guidance with the patient in either lateral decubitus or supine position on a traction table (Figure 2).

Given a quicker recovery, fewer wound complications, and better cosmesis in conjunction with a magnified field of view and the ability for dynamic evaluation of sciatic nerve kinematics, the endoscopic technique has gained increasing attention in recent years. So, the purpose of this systematic review was to assess the effectiveness of sciatic nerve decompression in the management of DGS by an endoscopic approach.

## Review

Methods

Search Strategy

A systematic review was conducted and reported according to the Preferred Reporting Items for Systematic Reviews and Meta-analyses (PRISMA) guidelines [23]. A comprehensive search of the PubMed, Web of Science, Cumulated Index to Nursing and Allied Health Literature (CINAHL), and SPORTDiscus databases were done from database inception until January 3, 2022. Both authors independently performed the search using the following terms: ("endoscopic" OR "endoscopy" OR "arthroscopy" OR "arthroscopic") AND ("Sciatic nerve decompression" OR "sciatic neurolysis" OR "sciatic nerve release" OR "deep gluteal syndrome" OR "piriformis syndrome" OR "piriformis release" OR "sciatic nerve entrapment").

Inclusion and Exclusion Criteria

All English-language clinical studies reporting the outcomes of sciatic nerve decompression of DGS by endoscopic approach were included for analysis. The exclusion criteria included biomechanical studies, cadaveric studies, review articles, conference abstracts, technical notes, case reports, or case series with fewer than five patients. When duplicate data were presented in two studies, data from the most recent study were included in the analysis. All titles and abstracts of the search results were reviewed for eligibility by both authors. Once finalized, the full-text articles and bibliography of the select articles were obtained, and further review was done independently. All discrepancies were resolved by discussion and reviewing the studies in detail.

Quality Assessment

The Coleman methodology score (CMS) [24] was utilized to independently assess the methodological quality of reporting of the selected articles by the investigators. This scoring system has 10 criteria divided into two parts, A (seven) and B (three). The total score ranges between 0 and 100, with 85 to 100 being excellent; 70 to 84, good; 55 to 69, fair; and less than 55 is classified poor. In part A, only one score is given for each of the seven sections, while the scores in part B are awarded for every option in each of the three sections if applicable.

Results

**Figure 3:**
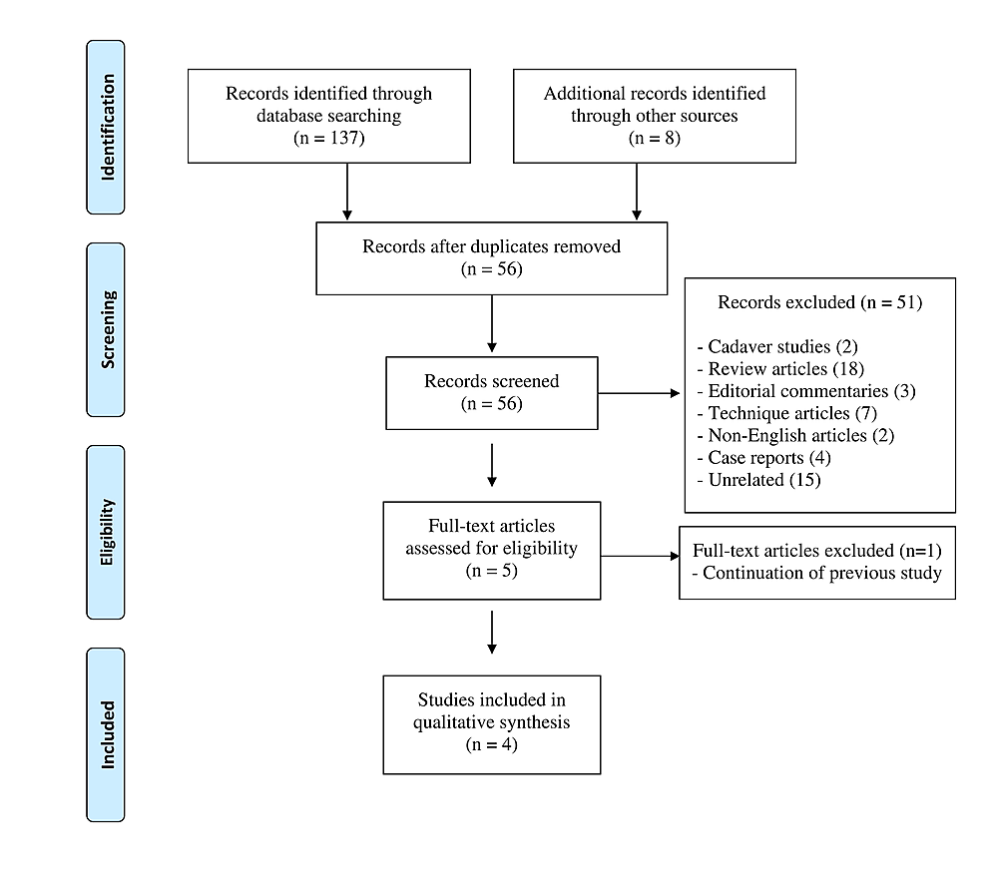
Flow diagram of the systematic review

The initial search had yielded a total of 145 articles for analysis. After the removal of 89 duplicates, 56 studies remained for further assessment. After a thorough screening, another 51 articles were excluded based on the pre-defined selection criteria resulting in five studies for potential inclusion. However, one more study needed exclusion that had the duplicate data from its previous study, thus making only four studies eligible for this systematic review. The flow diagram of search analysis is described in Figure 3.

Study Characteristics

One of the four finalized studies was a retrospective comparative study, and the other a prospective case series. The remaining two were retrospective case series (Table 1).

**Table 1:** Summary of study characteristics LOE: Level of evidence; DOS: Duration of symptoms

Authors	Year	Type of study (LOE)	No. of patients	Sex (M/F), n	Mean age (in years)	Mean DOS (in years)	Mean follow-up (in months)
Martin et al. [12]	2011	Retrospective case series (IV)	35	7/28	47 (20-66)	3.7 (1-23)	12 (6-24)
Ham et al. [25]	2018	Retrospective case series (IV)	24	11/13	47 (35-76)	1.0 (0.4-3)	32 (26-45)
Ilizaliturri et al. [26]	2018	Prospective case series (IV)	15	10/5	40.2 (28-50)	2.6 (1.5-4)	31.3 (18-50)
Park et al. [27]	2019	Retrospective comparative study (III)	70	39/31	50.3	1.9 (0.7-3)	30

A total of 144 patients were identified, consisting of 67 males and 77 females with a mean age of 46 years (range, 20 to 76 years). In the overall study population, the mean duration of symptoms from onset until surgery was 2.30 years (range, 0.4 to 23 years), and the mean follow-up was 26.3 months (range, six to 50 months).

Methodological Quality of the Studies

Regarding the quality evaluation, the mean CMS was 62 out of 100 points (range, 52 to 71), indicating that the overall quality of the included studies was at a ‘fair’ level. The score particulars for each of its 10 criteria of the individual studies are depicted in Table 2.

**Table 2:** Scores for each of the 10 criteria of the Coleman methodology score (CMS) for the included studies

	Methodology criterion	Martin et al. [12]	Ham et al. [25]	Ilizaliturri et al. [26]	Park et al. [27]	Mean
PART A	Study size: Number of patients (0–10)	4	4	0	10	4.5
	Mean follow-up in months (0–5)	2	5	5	5	4.3
	No. of surgical procedures/approaches (0–10)	10	10	10	10	10
	Type of study (0–15)	0	0	10	0	2.5
	Diagnostic certainty (0–5)	5	5	5	5	5
	Description of surgical technique (0–10)	10	10	10	10	10
	Description of postoperative rehabilitation (0–10)	0	5	10	5	5
PART B	Outcome criteria (0–10)	10	10	10	10	10
	Procedure of assessing outcomes (0–15)	6	6	6	6	6
	Description of subject selection process (0–10)	5	5	5	5	5
	Total Coleman methodology Sscore	52	60	71	66	62

Only one study ranked ‘good’ (score 71/100), and the rest included two ‘fair’ (60/100 and 66/100) and one ‘poor’ (52/100) quality study.

Presentation and Imaging Assessment

The DGS, among the selected studies, had a broad spectrum of clinical presentation. Most patients experienced chronic posterior hip pain accompanied by paraesthesias limiting their daily activities. The other symptoms included an inability to sit for at least 30 minutes, night pain, and back pain. Physical examination revealed recreation of buttock pain with one or more provocative tests, including Lasegue test; pace sign; flexion, adduction, internal rotation (FADIR) test; and seated piriformis test. Deterioration of motor function and foot drop were reported in a select group of patients after major trauma [27]. Plain radiographs, MRI examination, and diagnostic steroid injections were performed in all studies for preoperative assessment [12,25-27]. The EMG-NCS were performed selectively to exclude spinal pathology and other peripheral compressive sciatic neuropathies [27]. A summary of investigations, intraoperative particulars, outcomes, and complications are provided in Table 3.

**Table 3:** Investigations, intraoperative details, outcomes, and complications EMG-NCS: Electromyography-Nerve conduction study, mHHS: Modified Harris hip score: GT: Greater trochanter, MRI: Magnetic resonance imaging, MRA: Magnetic resonance arthrogram, VAS: Visual analog scale, HO: Heterotopic ossification, ABBM: Adhesion barrier bioabsorbable membrane

Authors	Investigations	Surgical technique	Etiology	Outcomes	Complications
Martin et al. [12]	Radiographs; MRA; Injection test	Supine, 2 cm or 3 cm portals; Release of causative structure(s)	Fibrous bands with thickened GT bursal tissue (n=27); piriformis tendon (n=18); obturator internus (n=3); hamstring tendon (n,2)	mHHS increased from 54.1 ± 13.1 to 78 ± 14.1; VAS score decreased from 6.9 ± 2 to 2.4 ± 2.6; 83% had no postoperative sciatic sit pain; 16 of 23 had good-to-excellent Benson symptom-rating scale	None
Ham et al. [25]	Radiographs; MRI/MRA; Injection test	Supine, 2 cm or 3 cm portals; Release of offending structure(s) and adhesion barrier bioabsorbable membrane (ABBM) to prevent re-adhesion	Fibrovascular bands (n,13); piriformis muscle and triceps coxae (n=8); ganglion (n=2); schwannoma (n=1)	mHHS increased from 59.4 ± 6.5 to 85.3 ± 8.3; P<0.001; VAS score decreased from 7.1 ± 0.9 to 2.5 ± 1.5; P<0.001; 87.5% excellent-to-good Benson symptom-rating scale	One recurrence due to foreign body reaction to ABBM salvaged by open decompression; One required conversion to open due to superior location of schwannoma
Ilizaliturri et al. [26]	Radiographs; MRI; Injection test	Lateral decubitus, 2 cm portals; Release of fibrous bands, bursa and piriformis tendon	Thickened GT bursa (n=14); fibrous bands (n=15); piriformis tendon entrapment (n=13)	mHHS increased from 46.8 ± 13.2 to 84.9 ± 4.7; VAS score decreased from 7.4 ± 0.7 to 1.86 ± 0.83; 93% excellent-to-good Benson symptom-rating scale	None
Park et al. [27]	EMG NCS; Radiographs; MRA; Injection test	Supine, combined hip arthroscopy and endoscopy of deep gluteal space, 2 cm or 3 cm portal; Release of offending structures	Trauma group (n=25): Perineural global scar tissue and fibrosis, focal fibrous scar bands with GT bursa, piriformis muscle, HO, acetabular screw; Idiopathic group (n=45): Fibrous bands, GT bursa, piriformis muscle, triceps coxae, quadratus, vascular, HO	Trauma group: mHHS increased from 61.5 ± 13.4 to 84.1 ± 8.1 (P=0.031); Idiopathic group: mHHS increased from 73.8 ± 10.3 to 94.4 ± 5.3 (P=0.003); VAS score decreased from 7.4± 1.5 to 2.6± 1.5 (P=0.001); Benson outcomes rating in trauma group was statistically lower than idiopathic group	No complete improvement in five patients of the trauma group that presented with foot drop

Surgical Management

The standard endoscopic technique described in the included studies consisted of a systematic inspection of peritrochanteric space through 2 cm to 3 cm portals. Fluoroscopy was utilized for portal placement and further intraoperative guidance. The sciatic nerve was inspected, and its kinematic excursion was assessed with various hip positions. The impinging structures on the nerve were identified and released using a combination of the blunt probe, scissors, shaver, and radiofrequency probe. Finally, dynamic testing of the nerve kinematics was repeated to ensure adequate mobility before closure. Except for one [22], all studies performed this endoscopic procedure in the supine position on the traction table using a 70-degree arthroscope via standard anterolateral, posterolateral, and auxiliary posterolateral portals. Ilizaliturri et al. employed lateral decubitus position (without traction) and used a 30-degree scope to enter the peritrochanteric space through proximal trochanteric and distal trochanteric portals [26]. Ham et al. placed an adhesive barrier bioabsorbable membrane (ABBM) over the nerve to minimize scar tissue formation [25]. Concomitant hip arthroscopy was performed by Park et al. before the exploration of the deep gluteal space for evaluation of any intra-articular pathologies [27].

Etiological Factors

Multiple etiological factors were identified during the endoscopic intervention of chronic DGS, and more than one cause was found to be responsible for sciatic nerve compression. Nonetheless, the most common cause was the presence of fibrovascular bands with hypertrophied trochanteric bursa, reported in 72% of patients. Sciatic nerve entrapment by musculotendinous structures was noticed in 44%, of which 84% were caused by piriformis alone. The mass effect due to a large ganglion was reported in two patients, while sciatic nerve schwannoma was detected in one. Sciatic nerve compression from the surrounding vascular structures was reported in three patients. Trauma to the sciatic nerve without any direct injury was described by Park et al. They presented a subgroup of traumatic sciatic neuropathies after pelvi-acetabular fractures or extensile open reconstructive hip surgeries and identified the presence of dense scar tissue formation around the nerve, causing widespread tethering and fibrosis. The heterotopic ossification (HO) mass enveloping the sciatic nerve was detected in two while one patient had nerve irritation due to the prominent tip of an acetabular screw [27].

Postoperative Rehabilitation

A detailed description of the rehabilitation protocol was mentioned in only one study [26]. Protected weight-bearing on the operated extremity was started the day after the surgery using crutches for two weeks. The hip flexion was limited to 90-degrees during the first week. A 30-degree limit was maintained for hip rotations and abduction for up to six weeks. Once patients achieved stable gait, therapy progressed for three to four months to regain strength and previous activities.

Postoperative Outcomes

All studies utilized validated outcome measures, including the modified Harris hip score (mHHS), visual analog scale (VAS) score, and the Benson outcomes questionnaire. Table 4 represents the scores of individual studies.

**Table 4:** Comparison of clinical outcome scores in individual studies of endoscopic decompression of the sciatic nerve mHHS: Modified Harris hip score, VAS: Visual analog scale, N/A: Not available, SF-12: 12-item short-form health survey score, PCS: Physical composite scale, MCS: Mental composite scale

	mHHS			VAS			Benson scale	SF-12
	Preoperative	Postoperative	p-value	Preoperative	Postoperative	p-value		
Martin et al. [12]	54.1 ± 13.1; (25.3 to 79.2)	78 ± 14.1; (44 to 100)	N/A	6.9 ± 2	2.4 ± 2.6	N/A	70% excellent-to-good	N/A
Ham et al. [25]	59.4 ± 6.5	85.3 ± 8.3	<0.001	7.1 ± 0.9	2.5 ± 1.5	<0.001	87.5% excellent-to-good	N/A
Ilizaliturri et al. [26]	46.8 ± 13.2; (21–78)	84.9 ± 4.7; (78-96)	<0.05	7.4 ± 0.7; (6-9)	1.86 ± 0.83; (1-4)	<0.05	93% excellent-to-good	N/A
Park et al. [27] Trauma group vs. Idiopathic group	61.5 ± 13.4; (32.5-72.8)	84.1 ± 8.1	0.031	N/A	N/A	N/A	56% excellent-to-good	PCS 41.1± 7.9; MCS 42.5±6.4 (P=0.030)
	73.8 ± 10.3; (55.6-83.4)	94.4 ± 5.3	0.003	7.4± 1.5	2.6± 1.5	0.001	84% excellent-to-good	PCS 46.8± 5.3; MCS 42.5±6.1 (P=0.580)

At a mean follow-up of 12 months, Martin et al. reported a mean mHHS gain of 23.9 and a mean VAS reduction of 4.5 with 70% of their patients achieving excellent-to-good scores according to the Benson outcomes questionnaire [12]. Ham et al. observed that the average postoperative gain in mHHS was 25.9 (p<0.001), and the drop in VAS was 4.6 (p<0.001), indicating significant improvement after endoscopic sciatic nerve decompression. Also, excellent-to-good Benson ratings were seen in 87.5% of their cases [25]. Likewise, Ilizaliturri et al. presented statistically significant postoperative improvement with a mean gain of 38.1 for mHHS (p<0.05) and a mean reduction of 5.5 for VAS (p<0.05) besides 93% of subjects demonstrating excellent-to-good Benson outcomes [26]. Finally, Park et al. noted a variation in the outcome measures between their major trauma and the idiopathic DGS patient groups. Statistically significant lower Benson outcomes rating (p=0.03) was observed in the major trauma cohort than the idiopathic group. Similarly, the mean gain in the mHHS and SF-12 scores for the major trauma group patients was considerably lower than that of the idiopathic group. Overall, less favorable outcomes were observed in the major trauma DGS group. All five patients of the same group that presented with foot drop failed to achieve complete neurological improvement due to intraneural fibrosis of the sciatic nerve [27].

Complications

Two unsatisfactory outcomes were reported [25] by Ham et al. One patient developed recurrent symptoms of sciatic nerve entrapment due to a strong foreign body reaction to the bioabsorbable barrier membrane that was applied to prevent re-adhesions. The patient subsequently underwent a second endoscopic surgery which required conversion to a formal open procedure because of extensive adhesions around the sciatic nerve. Similar conversion to open surgery was necessary for the other patient with schwannoma situated too proximal to be safely managed by endoscopic technique.

Discussion

This systematic review, with available evidence, has shown that endoscopic sciatic neurolysis is an effective treatment for DGS. At the 26.3-month follow-up, 80% of patients demonstrated excellent-to-good Benson ratings postoperatively. Though few studies are available with moderate quality, there was a significant improvement in postoperative clinical and functional outcomes with an extremely low rate of complications.

Deep gluteal syndrome is an important yet rare condition often presenting with non-specific symptoms of considerable overlap with that of adjacent pelvic, hip, sacroiliac, and spine pathologies. Awareness of these differences and myriad potential causes is critical to narrow the differential diagnosis. Precise palpation of anatomical structures and a combination of special examination maneuvres could enhance the clinical diagnostic yield [6,11]. Over the years, with the development and generous application of high-resolution imaging techniques in conjunction with rapid progress in the field of hip arthroscopy, much has changed in the management approach to DGS. Our systematic review identified fibrovascular bands with trochanteric bursal tissue as the most common etiological factor for this extra-spinal sciatic nerve entrapment followed by external rotator muscles in which piriformis is the prime pain generator.

Although MRI is regarded as the imaging modality of choice, musculoskeletal ultrasonography has excelled in recent decades. The US offers excellent soft-tissue visualization of deep gluteal space, the ability to perform a dynamic assessment of sciatic nerve kinematics, and allows side-to-side comparisons [1,28,29]. The US-guided perineural injections could be indispensable in many uncertain diagnostic situations besides therapeutic relevance [15,16]. All the selected studies in our review had utilized this imaging modality as a preoperative tool before subjecting to endoscopic neurolysis. Nevertheless, the US is highly operator-dependent and not precisely reproducible. Also, patient characteristics could influence the accuracy of US as the one with excessive soft tissues may pose difficulty in visualization.

The endoscopic approach for sciatic nerve decompression of DGS is a rapidly evolving field of minimally invasive surgery that requires substantial experience with general hip arthroscopic techniques. Thorough knowledge of endoscopic anatomy, meticulous preoperative planning, optimal and strategic placement of portals, generous integration of intraoperative fluoroscopy, step-wise systematic examination of the entire deep gluteal space, and awareness of potential iatrogenic complication are of paramount importance to ensure a safe and effective endoscopic procedure. That being said, conventional open surgery should still be reserved for cases with inadequate endoscopic exposure and revision situations addressing recurrences. Ham et al. recommended a primary open approach for a sciatic nerve schwannoma where the principal lesion is the nerve itself to minimize neurological complications [25]. Park et al. reported less promising postoperative outcomes in the selected subset of DGS patients with a history of fracture or extensile reconstructive surgery of the acetabulum that demonstrated global scar tissue formation and widespread tethering of the sciatic nerve [27]. Apart from the studies included in our review, there were a few case studies on endoscopic sciatic neurolysis. Hwang et al. treated a 42-year-female with a perineural cyst on the sciatic nerve causing symptoms of piriformis syndrome by arthroscopic decompression with no recurrence at 20 months follow up [7]. Yoon et al. described successful endoscopic resection of the tip of acetabular screw causing sciatic nerve irritation after revision total hip arthroplasty [10]. Postoperative recurrence due to the development of an infected hematoma after endoscopic sciatic neurolysis was reported in a 24-year-male that required open surgical evacuation and six weeks of postoperative antibiotics [8]. Our review analysis showed only one case of postoperative recurrence due to a foreign body reaction that was salvaged by open surgical decompression [25]. 

Limitations

The main limitation was the absence of high-quality evidence in publications dealing with the effects of endoscopic sciatic neurolysis of DGS. Only studies with evidence of level III and level IV were available, and overall, the methodological quality was moderate. Also, this systematic review included a small number of studies, which were further limited by small sample sizes, variable methodological quality, and significant heterogeneity in study designs and interventions, notably the practice of concomitant hip arthroscopy by only one study group. Finally, the selection bias was likely since the study population in one outnumbered the rest. Despite these limitations, we believe that relevant conclusions could be drawn from our study analysis, which applies to the current clinical practice of DGS. 

## Conclusions

Through this systematic review, we have explored the literature and identified a limited number of studies that evaluated the effectiveness of endoscopic sciatic nerve decompression for the surgical management of DGS. While the quality of evidence was modest at best, overall positive clinical outcomes could be observed among the studies with a meager rate of complications. Yet, high-quality randomized control trials with a large number of participants are necessary to draw definitive conclusions.
